# Stewart's Bedding

**Published:** 1915-03-13

**Authors:** 


### Institutional Needs.

### STEWART'S BEDDING.

An inspection of various institutions, particularly of those under the Poor Law, shows that many authorities still fail to realise the importance of having the highest grade bedding, notwithstanding the official warnings against the evils of flock which have been published in reports and circulars issued by the Local Government Board. There is, or should be, a hospital standard in these matters, and in placing contracts a firm should be sought which has specialised in meeting institutional requirements. D. Stewart & Co., 16 Staining Lane, Gresham Street, London, E.C., have devoted much attention to the subject of hospital bedding, towelling, waterproof sheeting, flannels, table linen, and, indeed, furniture. Hospital committees, Boards of Guardians, and sanatoria authorities should give their catalogue close attention, since the firm has had large experience in carrying out institutional contracts, both in London and in the provinces, and is in a position to meet the demands of hospital managers. An examination of their work is well worth while by all authorities with tenders to place for bedding and kindred materials.

